# Cardiac Metastasis Presenting As Right Ventricular Outflow Obstruction

**DOI:** 10.7759/cureus.49720

**Published:** 2023-11-30

**Authors:** Isaac Alsallamin, Lama Quraiba, Sameer Mtour, Afnan Alsallamin

**Affiliations:** 1 Clinical Medicine, Northeast Ohio Medical University, Cleveland, USA; 2 Internal Medicine, St. Vincent Charity Medical Center, Cleveland, USA; 3 Internal Medicine, Case Western Reserve University School of Medicine, Cleveland, USA; 4 Internal Medicine, University Hospitals Cleveland Medical Center, Cleveland, USA; 5 Medicine, Alfaisal University College of Medicine, Riyadh, SAU; 6 Cardiology, Al-Makassed Hospital, Jerusalem, PSE

**Keywords:** cancer, cardiac metastasis, arrhythmia, cardiac arrhythmia, ventricular tachycardia, right ventricle outflow tract obstruction, metastasis, squamous cell carcinoma

## Abstract

Right ventricular outflow tract obstruction (RVOTO) is characterized by an increased systolic pressure gradient between the right ventricle (RV) and the pulmonary artery. This rare condition can be diagnosed via echocardiography and may arise from various causes, including cardiac masses, pulmonary atresia with a ventricular septal defect, tricuspid valve thrombus, graft or wire calcification, or a cardiac tumor. We present the case of a 73-year-old male who was hospitalized after a syncope episode. Telemetry detected ventricular arrhythmia. Imaging identified a mass compressing the RV, causing RVOTO. A biopsy of the mass confirmed it as squamous cell carcinoma, which likely originated from the lung as a distant metastasis.

## Introduction

Right ventricular outflow tract obstruction (RVOTO) is identified by an echocardiogram when there is an increased systolic pressure gradient between the right ventricular outflow tract (RVOT) and the main pulmonary artery. Pressures above 25 mmHg can lead to arrhythmias and hemodynamic instability [1,2]. If the echocardiogram is inconclusive, a right-side cardiac catheterization is the next diagnostic step [1-5]. Elevated RVOT pressure can decrease blood flow in the pulmonary arteries, reducing oxygenation and affecting the left ventricle. This condition can compromise coronary blood flow in patients with coronary artery disease, risking ischemia and myocardial infarction and potentially leading to serious arrhythmias [1,3,4]. This case discusses a 73-year-old man with a history of cardiac and pulmonary ailments who presented with syncope and was found to have a right ventricular obstruction due to a suspected malignant mass, leading to hemodynamic instability.

## Case presentation

A 73-year-old man presented to the emergency department after experiencing syncope. While gardening, he felt lightheaded and attempted to sit but lost consciousness and fell, sustaining facial abrasions. He spontaneously regained consciousness within seconds, without post-syncope confusion.

On admission, his vitals were as follows: temperature of 36.7°C, heart rate of 57 beats per minute in sinus rhythm, respiratory rate of 15 breaths per minute, blood pressure 140/90 mmHg, and oxygen saturation of 95% while receiving 2 liters/minute via a nasal cannula. He had a facial abrasion but no significant open wounds. A cardiovascular examination revealed a regular pulse, normal first and second heart sounds and left lower parasternal holosystolic murmurs. A pulmonary examination showed decreased air entry on the right lower side, accompanied by rhonchi and intermittent wheezes. The left lung was clear upon auscultation. The abdomen was soft, non-tender, and non-distended, with no signs of hepatosplenomegaly. Examination of the upper extremities showed warmth and adequate perfusion, but there was redness and tenderness at a recent peripheral intravenous site on the left cubital fossa. The lower extremities were warm, and well-perfused, with symmetrical 2+ pulses and no edema.

The patient had a history of hypertension, hyperlipidemia, coronary artery disease, and ST-elevation myocardial infarction, which was managed with percutaneous coronary intervention to the left anterior descending artery (LAD). He also had a multinodular goiter, leading to subclinical hyperthyroidism. He was a former smoker with a 20-pack-year history but had quit for 30 years.

His home medications included amlodipine 5 mg daily, aspirin 81 mg daily, atorvastatin 80 mg daily, and levothyroxine 50 µg daily. Further symptom review revealed a 15-pound unintentional weight loss in recent months, recurrent syncope, and dizziness. However, he denied experiencing fever, appetite loss, blurry vision, rashes, shortness of breath, orthopnea, paroxysmal nocturnal dyspnea, constipation, abdominal pain, bone pain, night sweats, or lower limb swelling. Table 1 presents his initial laboratory findings.

**Table 1:** Initial laboratory investigations Other than leukocytosis, the labs were insignificant.

Investigation	Result
White blood cell count	19.6 x10^9/L
Neutrophils	16.3 x10^9/L
Monocytes	1.5 x10^9/L
Hemoglobin	14.5 g/dL
Platelets	20 x10^9/L
Sodium	138 mmol/L
Potassium	4.4 mmol/L
Creatinine	1.2 mg/dL
Pro-B-type natriuretic peptide	237 pg/mL
High-sensitivity troponin	17 ng/L (negative)
Protein	8.4 g/dL
Albumin	4.1 g/dL
Thyroid-stimulating hormone	0.5 mIU/L
Free thyroxine	1.45 ng/dL
Prostate-specific antigen	0.85 ng/mL
Glycated hemoglobin	5.4%
Low-density lipoprotein	57 mg/dL

An ECG obtained by emergency medical services showed a normal sinus rhythm followed by a wide complex tachycardia with a left bundle branch block morphology.

**Figure 1:**
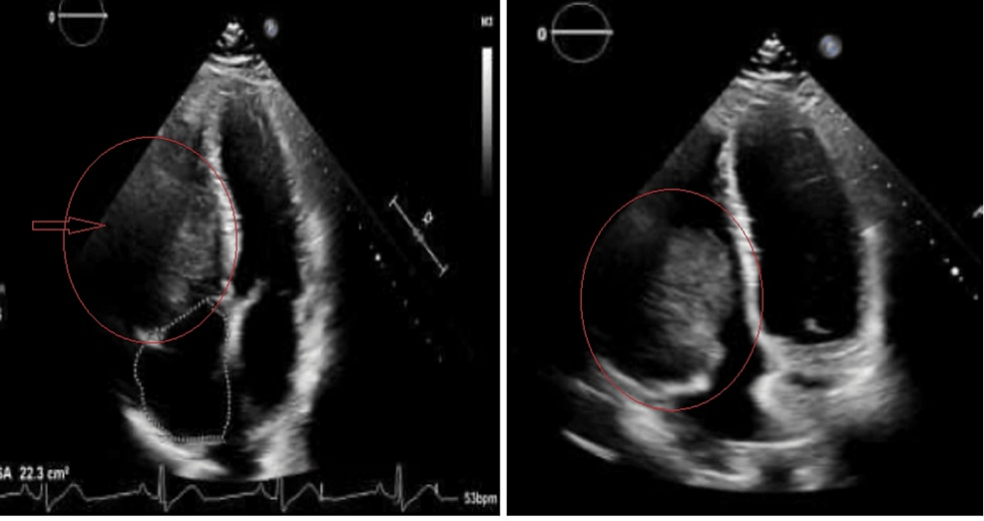
Echocardiogram showing a right ventricular mass

Considering the clinical presentation and ECG results, the patient underwent coronary catheterization. No culprit lesion was identified in the catheterization lab; the previously stented LAD was patent, and the first obtuse marginal branch had 70% occlusion. The percutaneous coronary intervention was not performed. The patient received amiodarone 150 mg twice daily due to suspected ventricular arrest, although no related rhythm was documented pre-admission. Echocardiography indicated a normal left ventricular ejection fraction of 55% to 60% with no wall motion abnormalities and a large mass (approximately 6.9 cm x 5.2 cm) in the right ventricular (RV) free wall from the base to the mid-right ventricle (Figure 1).

During hospitalization, telemetry detected slow, monomorphic ventricular tachycardia (VT). The patient was treated with lidocaine and amiodarone, and lidocaine was continued for suspected ischemia-induced VT.

**Figure 2:**
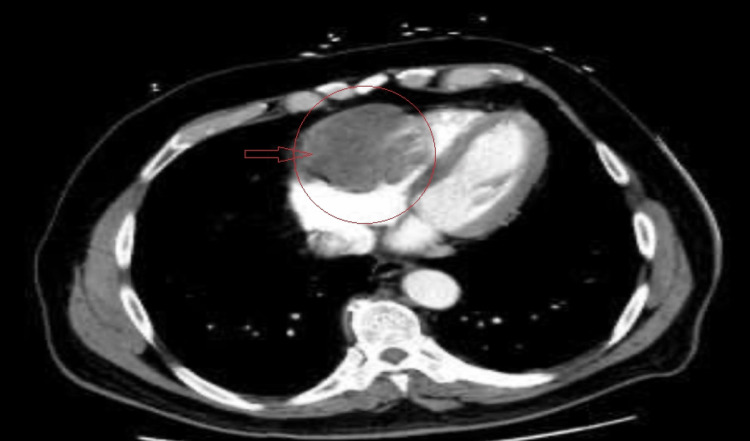
The chest CT scan shows a right ventricular mass

A chest CT scan revealed a 2 cm mass in the right middle lung lobe adjacent to the pericardium and existing thyroid nodules (Figure 2).

**Figure 3:**
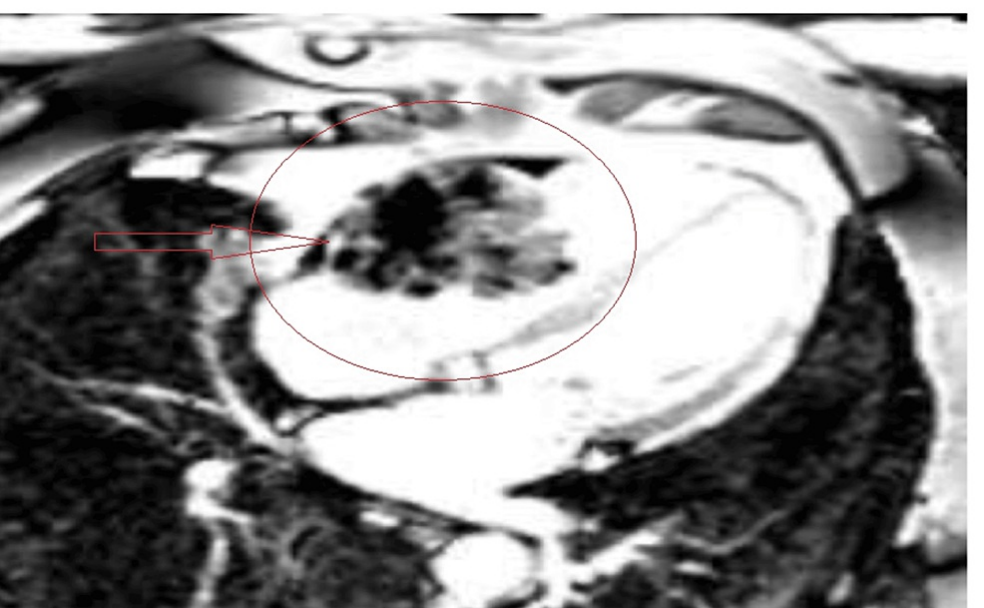
CT with contrast showed a 3 cm irregular mass in the right middle lobe, abutting the pericardium, several subcentimeter nodules, and no lymphadenopathy.

A full-body CT with contrast revealed a 3 cm irregular mass in the right middle lobe, abutting the pericardium, several subcentimeter nodules, and no lymphadenopathy (Figure 3).

**Figure 4:**
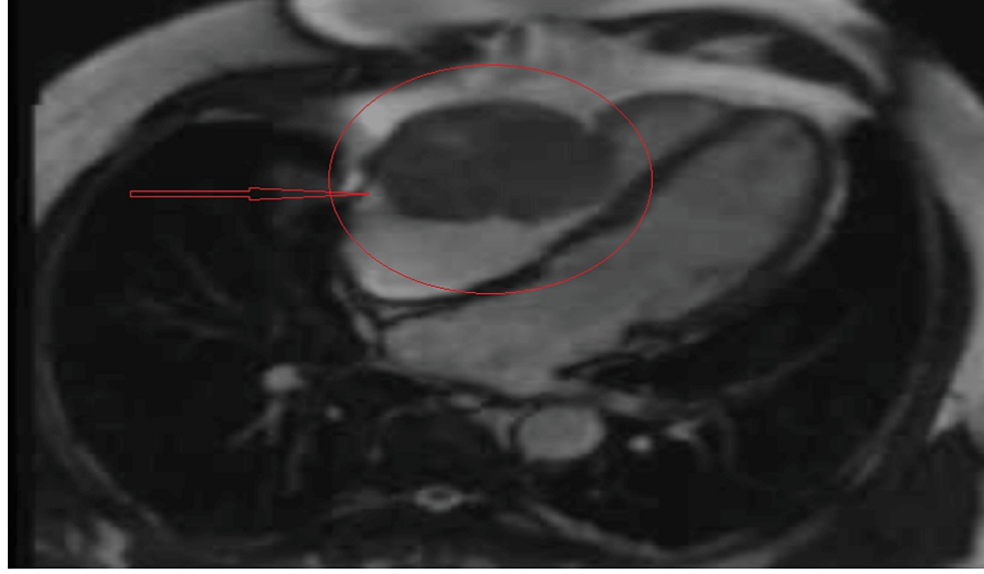
The cardiac MRI shows a right ventricular mass

Malignancy with potential metastasis became the primary consideration. Cardiac magnetic resonance imaging (cMRI) and a positron emission tomography (PET) scan were ordered. The cMRI showed a heterogeneous mass consistent with malignancy, possibly metastatic from a known right lung mass (Figure 4).

**Figure 5:**
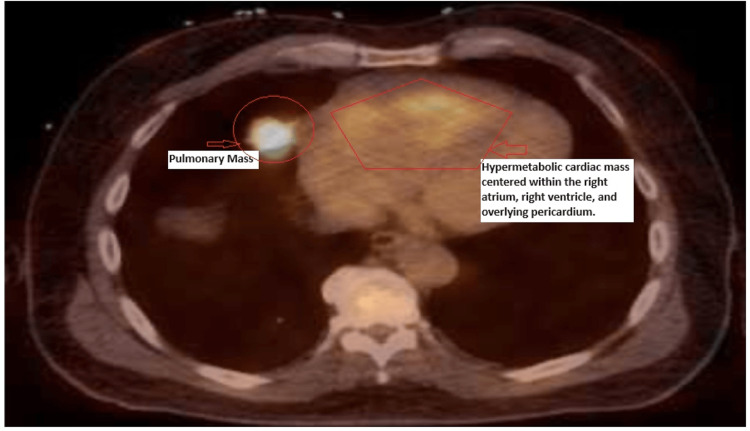
The PET scan shows a markedly hypermetabolic pulmonary mass in the right middle lobe measuring 3.2 x 2.6 cm, which is consistent with malignancy, and a hypermetabolic cardiac mass centered within the right atrium, right ventricle, and overlying pericardium are seen. The findings are consistent with a malignant cardiac lesion. PET: positron emission tomography

The PET scan identified hypermetabolic masses in the right middle lobe and cardiac regions, consistent with malignancy (Figure 5).

He was administered antiarrhythmic drugs due to concerns about VT but developed sinus bradycardia with a rate of 55 to 60 beats per minute. Lidocaine was discontinued, and amiodarone was reintroduced. The patient also developed swelling, tenderness, and redness in the left cubital region. Ultrasound of the left upper extremity revealed a thrombosed superficial vein and a small abscess, which was subsequently drained and treated with antibiotics.

A right-sided heart catheterization with a cardiac biopsy was performed. During the procedure, one episode of VT arrest occurred. Biopsy samples showed myocardial tissue with mild fibrosis. Consultation with pulmonologists led to a bronchoscopy and a CT-guided lung biopsy of a suspicious right middle lobe lung mass. Results indicated non-small cell carcinoma, consistent with squamous cell carcinoma. The patient continued aspirin, statin, and oral amiodarone 400 mg. Both the oncology and palliative care teams were consulted to determine long-term treatment goals.

The biopsy results confirmed a diagnosis of lung squamous cell carcinoma. The patient had a follow-up visit with the palliative oncology department six months after discharge but did not return for further appointments.

## Discussion

Right ventricular outflow tract obstruction in adults can stem from various causes, including internal or external cardiac masses, mediastinal masses, lymph nodes, hematomas, abscesses, or complications after surgery that compress the RV [1-4,6]. Our patient's syncope stemmed from reduced preload due to the RV's compromised stroke volume, exacerbated by an obstructing mass and an episode of VT. Symptoms vary from dizziness and syncope to arrhythmias, intensified by dehydration, anxiety, and specific medications. Clinically, there may be a systolic murmur, hypovolemia, or shock. Other signs include a mediastinal mass or pericardial effusion [1,3-8]. Its management hinges on the cause. Physicians should avoid reducing preload in acute cases and be cautious with inotropes. Antiarrhythmics can address ventricular tachyarrhythmia with continuous hemodynamic monitoring, and sometimes treatments are palliative.

In reviewing the literature, there are few reported cases of RVOTO. Huang et al. reported a mesenchymal tumor diagnosed as an atypical capillary hemangioma, where surgical excision of the mass resulted in a favorable outcome [7]. Zeng et al. described two postoperative cases where RVOTO was complicated using vasopressors, advising against their use in such scenarios [8]. Kirshbom et al. documented RVOTO occurring two months post-lung transplantation, which was complicated by a ventricular septal defect (VSD) and right-sided heart failure. The surgical correction had an impressive prognosis and outcome [9]. Miyaji et al. encountered RVOTO in the postoperative intensive care unit due to disopyramide administration, recommending beta-blocker usage instead [10]. Ramakrishnan et al. discussed an RVOTO case where ablation with alcohol led to successful amelioration of RV pressure [11]. Shuntoh et al. reported on infective endocarditis leading to RVOTO due to a prolapsing aortic valve, resulting in an aneurysm of the aortic sinus of Valsalva and mild aortic regurgitation. This was managed by surgical closure of the aortic sinus fistula with continuous suture, excision of the aneurysmal sac, and VSD patch closure [12]. Lastly, Kurzidim et al. presented a case of RVOTO caused by cardiac metastasis of a plasmacytoma, treated with palliative radiotherapy, which reduced tumor size and improved symptoms; however, the patient ultimately succumbed to radiation-induced pneumonitis [13].

In our case, the EKG showed some nonspecific new EKG changes. The initial EKG showed some changes related to the mass location; chamber enlargement, strain, or evidence of ischemia or arrhythmia is the first clue. Echocardiography is one of the best screening tools for syncope and cardiac masses. It is sensitive, but not specific. It showed the RV mass and signs of RVOT, with an increased pressure difference across the pulmonary trunk. On the other hand, transesophageal echocardiography (TEE) gives clearer images, helps with the identification of mass location, and helps narrow down alternative diagnoses such as valve pathology or thrombus. Transesophageal echocardiography is an excellent tool to measure the pressure differences for better estimation of RVOT. Advanced imaging modalities, such as cMRI, can increase sensitivity, mass differentiation, and extension.

## Conclusions

Right ventricular outflow tract obstruction can lead to life-threatening events, necessitating a comprehensive cardiac evaluation. This evaluation includes ECG, cardiac echocardiography, and cMRI. Prompt intervention is essential to alleviate the pressure, whether the obstruction arises from a mass effect or is medication-induced. The primary treatment approach involves fluid resuscitation, avoiding inotropic agents, and using antiarrhythmic medications with caution.
